# Head to head comparisons of two modalities of perfusion adenosine stress echocardiography with simultaneous SPECT

**DOI:** 10.1186/1476-7120-7-19

**Published:** 2009-04-20

**Authors:** Petri Gudmundsson, Kambiz Shahgaldi, Reidar Winter, Magnus Dencker, Mariusz Kitlinski, Ola Thorsson, Lennart Ljunggren, Ronnie B Willenheimer

**Affiliations:** 1 Department of Biomedical Laboratory Science, Malmö University, Malmö, Sweden; 2 Department of Cardiology, Karolinska University Hospital Huddinge, Stockholm, Sweden; 3 Department of Clinical Physiology, Karolinska University Hospital Huddinge, Stockholm, Sweden; 4 Department of Clinical Physiology, Lund University, Malmö University Hospital, Malmö, Sweden; 5 Department of Cardiology, Lund University, Malmö University Hospital, Malmö, Sweden; 6 Department of Clinical Sciences, Medicine/Cardiology, Lund University, Malmö University Hospital, Malmö, Sweden; 7 Heart Health Group, Malmö, Sweden

## Abstract

**Background:**

Real-time perfusion (RTP) contrast echocardiography can be used during adenosine stress echocardiography (ASE) to evaluate myocardial ischemia. We compared two different types of RTP power modulation techniques, angiomode (AM) and high-resolution grayscale (HR), with ^99m^Tc-tetrofosmin single-photon emission computed tomography (SPECT) for the detection of myocardial ischemia.

**Methods:**

Patients with known or suspected coronary artery disease (CAD), admitted to SPECT, were prospectively invited to participate. Patients underwent RTP imaging (SONOS 5500) using AM and HR during Sonovue^® ^infusion, before and throughout the adenosine stress, also used for SPECT. Analysis of myocardial perfusion and wall motion by RTP-ASE were done for AM and HR at different time points, blinded to one another and to SPECT. Each segment was attributed to one of the three main coronary vessel areas of interest.

**Results:**

In 50 patients, 150 coronary areas were analyzed by SPECT and RTP-ASE AM and HR. SPECT showed evidence of ischemia in 13 out of 50 patients. There was no significant difference between AM and HR in detecting ischemia (p = 0.08). The agreement for AM and HR, compared to SPECT, was 93% and 96%, with Kappa values of 0.67 and 0.75, respectively (p < 0.001).

**Conclusion:**

There was no significant difference between AM and HR in correctly detecting myocardial ischemia as judged by SPECT. This suggests that different types of RTP modalities give comparable data during RTP-ASE in patients with known or suspected CAD.

## Background

Patients with suspected myocardial ischemia are often assessed using different types of exercise tests for ischemic evaluation, according to current clinical guidelines [1,2]. Exercise ECG is considered the first-line technique for assessment of ischaemia, whereas single-photon emission computed tomography (SPECT) or dobutamine atropine stress echocardiography (DSE) are suggested when exercise ECG are non-diagnostic or non-interpretable [3,4]. Both SPECT and DSE are more accurate methods than exercise ECG, although more expensive [4-7].

Adenosine stress echocardiography (ASE) is a less expensive technique as compared to SPECT and more tolerable compared to DSE for the assessment of patients with suspected coronary artery disease. However, ASE, using only wall motion evaluation, is less accurate for the detection of myocardial ischemia as compared to DSE and SPECT. [8-10]. The use of second-generation contrast agents in myocardial contrast echocardiography enables assessment of myocardial perfusion during ECG triggered image acquisition echocardiography with high mechanical index, i.e. harmonic power Doppler. This technique is not only technically demanding, but also has limitations, e.g. because no wall motion evaluation is possible.

Real-time myocardial perfusion echocardiography (RTP) has recently been developed using highly contrast specific, low mechanical index imaging techniques. RTP during intravenous infusion of a second-generation contrast agent allows for simultaneous analysis of myocardial perfusion and wall motion. Earlier studies with RTP have shown promising results for the evaluation of myocardial perfusion [11-18]. However, there is limited clinical data on the accuracy of RTP-ASE for the detection of myocardial ischemia in unselected patient groups. The RTP techniques continue to evolve and new improvements are developed continuously. There are, however, only few studies carried out to find out if the new developments really improve the evaluation of ischemia.

Adenosine is a sub-optimal stressor for wall motion analysis and may be less sensitive in detecting ischemia [3]. On the other hand, perfusion defects seem to be more visible with adenosine compared to dobutamine [9]. The myocardial contrast echocardiography technique is still burdened with perfusion artifacts and echocardiographic shadowing, which limit the number of interpretable myocardial segments. These segments can in most cases, using RTP, still be interpreted with wall motion analysis, which therefore increases the usefulness of RTP-ASE without diminishing accuracy considerably [15]. Thus, combining perfusion and wall motion assessment, RTP-ASE has the potential of being as accurate and feasible as DSE and SPECT, and may be a swift, bedside-accessible, useful decision-making tool for risk assessment of patients with suspected myocardial ischemia.

Two techniques of RTP power modulation have been developed. The first technique available was angio-mode (AM), in which echoes from contrast bubbles are displayed as colored pixels in the two dimensional images and are, therefore, easily differentiated from the tissue echoes, which are displayed in grey scale. The most recently developed power modulation technique is the high-resolution grey scale (HR). Using HR, the only echoes displayed are grey scale echoes from contrast bubbles. The HR technique is meant to suppress all echoes originated from tissue and has a higher spatial resolution compared to AM. The difference between the two techniques has to our knowledge not been examined by head to head comparison.

The aim of the present study was to compare the two power modulation techniques, AM and HR, during RTP-ASE, for the detection of myocardial ischemia, as judged by ^99m^Tc-tetrofosmin SPECT, in a clinical patient population with known or suspected myocardial ischemia.

## Methods

### Patient population

The patient population consisted of 51 randomly selected patients with known or suspected coronary artery disease, admitted to SPECT evaluation, to participate in the study. The patient's acoustic windows were not screened prior to inclusion. One of the included patients had non-interpretable echocardiography images, both regarding wall motion and perfusion, and was therefore excluded from the study. The institutional ethics committee of the Lund University, Sweden, approved the study (Lu 113-03). Written informed consent was obtained from all participating patients.

### Study protocol

#### Myocardial contrast echocardiography

The echocardiographic equipment used was a Sonos 5500 (Philips, Andover, MA, USA) with S3 probe and RTP using power modulation AM and HR. Patients were examined in a left lateral recumbent position. The second-generation contrast agent Sonovue^® ^was infused in the left decubital vein using an infusion pump dedicated for this purpose (VueJect^® ^Esaote, Genova, Italy; Bracco™, Milano, Italy), which automatically rotates the syringe to prevent sedimentation. The infusion rate of Sonovue^® ^was set between 1.0 and 1.3 ml/min [19]. Adenosine and echo contrast were infused in the same peripheral venous catheter, using a separate infusion pump through a three-way stopcock. Adenosine was given at an infusion rate of 100 μg/kg/min during one minute, and was thereafter increased to 140 μg/kg/min.

All 50 patients underwent RTP imaging (mechanical index = 0.1) during infusion of echo contrast, at rest and after a minimum of one minute of hyperemia during adenosine stress (at 140 μg/kg/min). Image acquisition was started after a minimum time of one minute of Sonovue^® ^infusion. RTP image loops containing 8–10 heartbeats were collected from the parasternal long- and short-axis and apical four- and two-chamber views, respectively. At the beginning of each loop a destruction impulse of 10 high mechanical index frames (mechanical index = 1.5) were given to destroy all contrast micro bubbles in the myocardium [20].

During RTP AM, the angio-mode gain was set at between 60 and 70%, depending on what was suitable for the individual patient as judged by a visual on-line assessment, and 2D grayscale gain was set at zero. During RTP HR the grey-scale gain was set between 90 and 95%, depending on what was suitable for the individual patient, as judged by a visual on-line assessment. Focus was set close to the base of the left ventricle. All images were stored digitally for later off-line analysis.

#### SPECT

The rest and stress studies were performed using a 2-day protocol, starting with injection of 600 MBq ^99m^Tc-tetrofosmin at stress. Stress was simultaneous with the RTP-ASE. Normal findings at stress were not followed by a rest study [21,22]. Pathological stress studies were followed by a rest study with injection of 800 MBq ^99m^Tc-tetrofosmin. A five-minute adenosine infusion protocol was used. Patients who had cardiac medications, which could interfere with the stress test, were informed to have their medication interrupted prior to the stress test. The decision whether to interrupt the drug administration was at the discretion of the referring physician. Starting the infusion with 100 μg/ml/min of adenosine for 1 minute, the dose was then increased to 140 μg/ml/min for two minutes before injecting ^99m^Tc-tetrofosmin. Infusion of adenosine was continued for 2 min after the injection of ^99m^Tc-tetrofosmin. The scintigraphic data were acquired one hour after the end of the stress test, using continuous SPECT over 180 degree elliptical rotation from the 45 degree right anterior oblique position, with a dual-head gamma camera (Siemens AG Medical Solutions, Erlangen, Germany). Low energy high-resolution collimator and a zoom factor of 1.0 were used. We obtained 64 projections in a 128 × 128 matrix, with an acquisition time of 20 s per projection. Tomographic reconstruction and calculation of short axis slice images were performed using Siemens software. A two-dimensional Butterworth pre-reconstruction filter was used with critical frequency of 0.35, order 5. For each patient, the same sets of short axis slices were then processed with an automatic software package (4D-MSPECT) on a Siemens e.soft workstation. The software package defined apex and base and generated, coronal, longitudinal, sagital tomographic slices as well as polar maps with schematic map of the territories of the main coronary arteries used for scoring. Radiotracer uptake of the vascular segments was scored visually and stress images were compared with rest images regarding ischemia and infarct. The specialist in nuclear medicine who performed the scoring was blinded to the results of the RTP analysis.

#### RTP-ASE image interpretation

Image interpretation was performed off line, analyzing myocardial perfusion and wall motion by RTP-ASE, using the EnConcert Image Diagnosis Application (Philips, Andover, MA, USA). A separate analysis exclusively of perfusion was also made for both AM and HR to estimate the value of sole perfusion analysis. AM and HR were evaluated on separate occasions blinded to one another and blinded to the result of the SPECT interpretation.

**Figure 1:**
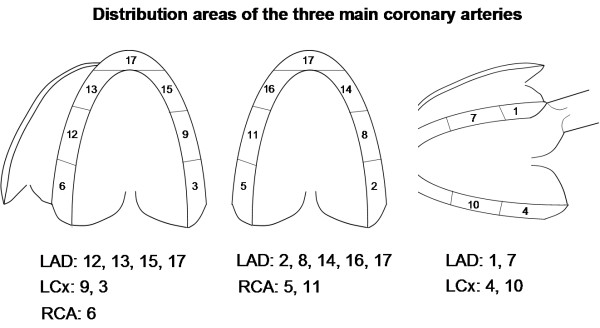
**Distribution territories of the three main coronary arteries**. Distribution territories of the three main coronary arteries in a 17 segment model. Left anterior descending (LAD), left circumflex (LCx) and right coronary artery (RCA).

Each segment was attributed to one of the three main coronary vessel areas of interest; the left anterior descending (LAD); the left circumflex (LCx); and the right posterior descending (RPD) (see Figure 1). Myocardial ischemia was visually evaluated comparing rest and stress images, using both perfusion and wall motion analysis in a complementary manner. A visually detected perfusion defect during stress was used as the principal marker of ischemia. Thus, a myocardial segment was considered ischemic if perfusion was impaired in the stress images, compared to the rest images [9]. Perfusion defects were analyzed at the earliest four beats following the destruction impulse at rest and after two beats at peak stress.

Wall motion was used in addition to reveal perfusion defect artifacts at rest and to evaluate segments with suspected perfusion artifacts at stress. Since wall motion should not be normal if a segment has a true perfusion defect at rest, a perfusion defect at rest was considered to be an artifact when wall motion was normal in that segment. A perfusion defect at peak stress was considered to be an artifact if there was a suspicion of a perfusion artifact, such as lateral or anterior shadowing from ribs or lungs, or basal segments shadowed by contrast. In such segments, the ischemic evaluation was based on wall motion analysis alone. If wall motion decreased at stress compared to rest images, the segment was considered ischemic. Since perfusion can be decreased without a decrease in wall motion in ASE, the use of solitary wall motion analysis in segments with perfusion artifacts might decrease the sensitivity with regard to ischemia. However, this complementary use of wall motion analysis increases the number of interpretable segments without negatively affecting specificity [11].

### Statistical analysis

Power calculation for the RTP-ASE comparison to SPECT was based on a sensitivity and specificity between 80 and 90% of the methods used. We assumed a sensitivity and a specificity of 85% in the study. With 50 patients we would have a 95% confidence interval of ± 10% around sensitivity and specificity. The SPSS^® ^(Version 12.0.1, Chicago, IL, USA) statistical program was used for the statistical analysis. We calculated sensitivity and specificity, positive and negative predictive values (PPV, NPV), as well as accuracy and Kappa values in the three predefined distribution areas of the three main coronary vessels. To assess differences between AM and HR, the chi-squared test was performed. Method of reference for the ischemia evaluation in the study was the presence or absence of reversible ischemia in the SPECT examination. Results are expressed as mean ± SD and as percent. P < 0.05 denoted significance.

## Results

Baseline characteristics including clinical data extracted from patient's records are shown in Table 1. Mean age of the 50 patients was 70 years, two thirds were women and mean left ventricular ejection fraction was close to normal. A history of previous myocardial infarction was found in less than half of the patients, around one third had undergone previous coronary intervention, a majority had no previous hospitalization or intervention due to coronary artery disease, and around one fifth of the patients had no cardiac medication. At SPECT, 13 (26%) of the patients were ischemic in at least one coronary area.

**Table 1:** Patients characteristics, including clinical data extracted from patient's records.

Age	70 (± 8)
Male	36%
LVEF at rest	55 (± 9) %
Previous AMI	40%
Previous PCI	22%
Previous CABG	16%
Heart failure	12%
Hypertension	54%
Valvular surgery	0%
Beta-blocker	54%
ACE inhibitor	28%
ARB	14%
Nitro-glycerin (short acting)	60%
Nitrates (long acting)	28%
Diuretics	29%
Calcium blocker	24%
Sinus rhythm	92%
Dilated left ventricle	10%
Dilated left atrium (n = 29)	31%
Significant valvular disease (n = 31)	6%
Regional WMA/PD at rest	60%

LVEF, left ventricular ejection fraction; AMI, acute myocardial infarction; PCI, percutaneous coronary intervention, CABG, coronary artery bypass grafting; ACE, angiotensin converting enzyme; ARB, angiotensin-receptor blocker, WMA, wall motion abnormality; PD, perfusion defect.

**Figure 2:**
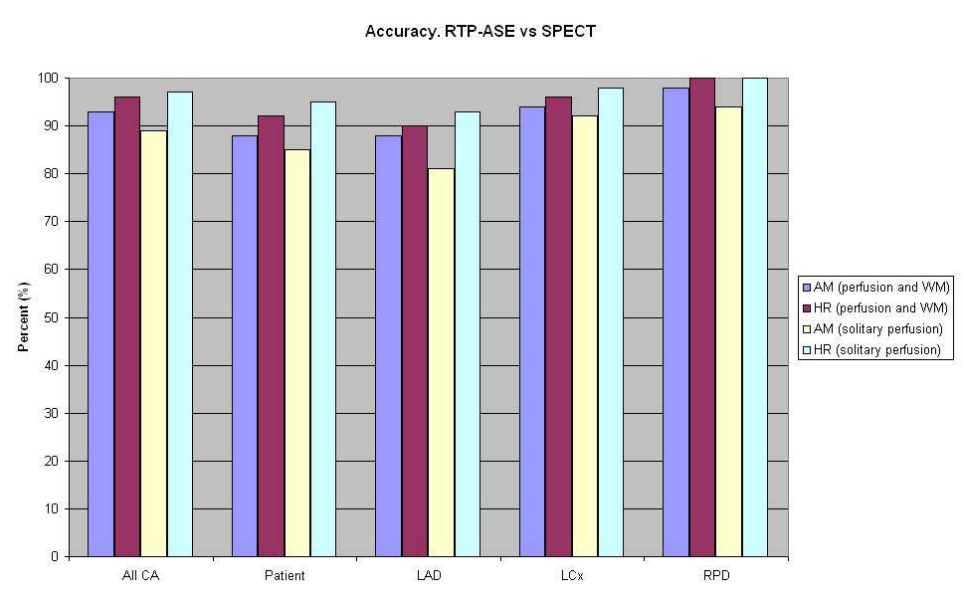
**A graph of accuracy between different modalities of RTP-ASE versus SPECT**. Left anterior descending (LAD), left circumflex (LCx) and right coronary artery (RCA). Angio-mode (AM), high resolution (HR) and wall motion (WM).

Of 150 coronary areas assessed, all were considered interpretable and were analyzed both using SPECT and RTP with AM and HR. The overall level of agreement between RTP-ASE and SPECT in detecting ischemia was 93% for AM and 96% for HR. The chi-square test for difference between AM and HR in correctly judging ischemia was borderline-significant (p = 0.08). The Kappa values were 0.67 for AM and 0.75 for HR (p < 0.001). Accuracy, sensitivity, specificity, predictive values and Kappa values for the detection of myocardial ischemia of RTP-ASE AM and HR, in the respective coronary areas, compared to SPECT are shown in Table 2. HR generally showed somewhat higher values for kappa, accuracy, positive prediction and specificity, whereas AM showed higher sensitivity values. The NPVs were similar. The same comparative values for the sole perfusion interpretation concerning AM and HR compared to SPECT are displayed in Table 3, demonstrating differences similar to the combined perfusion and wall motion analysis. Accuracy and Kappa values for the agreement between AM and HR are shown in Table 4, indicating lesser agreement between AM and HR than for their respective comparison with SPECT. Figure 2 represents a graph of accuracy between different modalities of RTP-ASE versus SPECT.

**Table 2:** RTP-ASE angio-mode (AM) versus high resolution (HR) with combined perfusion and wall motion analysis.

		All CA (n = 150)	Patient (n = 50)	LAD (n = 50)	LCx (n = 50)	RPD (n = 50)
**AM**	Accuracy (%)	93	88	88	94	98
**AM**	PPV (%)	57	71	54	50	100
**AM**	NPV (%)	99	97	100	100	98
**AM**	Sensitivity (%)	92	92	100	100	75
**AM**	Specificity (%)	93	87	86	94	100
**AM**	Kappa	0.67***	0.72***	0.63***	0.64***	0.85***
**HR**	Accuracy (%)	96	92	90	96	100
**HR**	PPV (%)	77	91	67	67	100
**HR**	NPV (%)	98	92	93	98	100
**HR**	Sensitivity (%)	77	78	57	67	100
**HR**	Specificity (%)	98	97	95	98	100
**HR**	Kappa	0.75***	0.78***	0.56***	0.65***	1.00***

Accuracy, positive (PPV) and negative (NPV) predictive values, sensitivity, specificity and Kappa of RTP-ASE using SPECT as method of reference.

CA, coronary area; LAD, left anterior descending coronary artery; LCx, left circumflex artery; RPD, right posterior descending coronary artery, *** = p < 0.001.

**Table 3:** RTP-ASE angio-mode (AM) versus high resolution (HR) using solitary perfusion analysis.

		Any CA (n = 134)	Patient (n = 39)	LAD (n = 44)	LCx (n = 42)	RPD (n = 48)
**AM**	Accuracy (%)	89	85	81	92	94
**AM**	PPV(%)	48	71	43	50	60
**AM**	NPV (%)	99	96	100	100	98
**AM**	Sensitivity (%)	92	92	100	100	75
**AM**	Specificity (%)	89	81	78	91	96
**AM**	Kappa	0.58***	0.68***	0.50***	0.63***	0.63***
**HR**	Accuracy (%)	97	95	93	98	100
**HR**	PPV (%)	80	90	67	100	100
**HR**	NPV (%)	98	97	97	98	100
**HR**	Sensitivity (%)	80	90	80	50	100
**HR**	Specificity (%)	98	97	95	100	100
**HR**	Kappa	0.78***	0.87***	0.69***	0.66***	1.00***

Accuracy, positive (PPV) and negative (NPV) predictive values, sensitivity, specificity and Kappa of RTP-ASE using SPECT as method of reference.

CA, coronary area; LAD, left anterior descending coronary artery; LCx, left circumflex artery; RPD, right posterior descending coronary artery, *** = p < 0.001.

**Table 4:** Agreement between RTP-ASE angio-mode and high resolution.

	Any CA (n = 150)	Patient (n = 50)	LAD (n = 50)	LCx (n = 50)	RPD (n = 50)
**RTP + WM**					
Agreement (%)	92	80	82	94	98
Kappa	0.61***	0.51***	0.43**	0.64***	0.85***

**Sole RTP**					
Agreement (%)	88	77	78	91	94
Kappa	0.47***	0.52**	0.43**	0.37**	0.54***

Combined perfusion and wall motion analysis, and using solitary perfusion analysis.

CA, coronary area; LAD, left anterior descending coronary artery; LCx, left circumflex artery; RPD, right posterior descending coronary artery; RTP, real time perfusion; WM, wall motion; ** = p < 0.01; *** = p < 0.001.

In Table 5, the numbers of non-interpretable coronary areas are presented. In the sole perfusion analysis the numbers include coronary areas considered to be perfusion artifacts. There is evidence of similar loss of interpretable coronary areas for both AM and HR.

**Table 5:** Non-interpretable coronary areas for RTP-ASE angio-mode (AM) and high resolution (HR).

		Any CA (n = 150)	Patient (n = 50)	LAD (n = 50)	LCx (n = 50)	RPD (n = 50)
**AM**	RTP + WM (%)	0	0	0	0	0
**AM**	Sole RTP (%)	13.3	22.0	16.0	24.0	0
**HR**	RTP + WM (%)	0	0	0	0	0
**HR**	Sole RTP (%)	10.7	22.0	12.0	16.0	4.0

Combined perfusion and wall motion analysis, and solitary perfusion analysis.

CA, coronary area; LAD, left anterior descending coronary artery; LCx, left circumflex artery; RPD, right posterior descending coronary artery; RTP, real time perfusion; WM, wall motion.

Inter- and intra-observer agreements for the interpretation of ischemia according to the RTP-ASE AM and HR examinations are presented in Table 6.

**Table 6:** Inter and intra observer agreement of myocardial contrast echocardiography ischemia interpretation.

	Total	LAD	LCx	RPD
Inter-observer (%) AM	91	88	97	88
Kappa	0.72***	0.73***	0.90***	0.28 ns

Intra-observer (%) AM	94	94	91	97
Kappa	0.76***	0.84***	0.62***	0.78***

Inter-observer (%) HR	95	95	95	95
Kappa	0.70***	0.64***	0.64***	0.78***

Intra-observer (%) HR	97	95	95	100
Kappa	0.78***	0.64**	0.64	1.00***

RTP-ASE angio-mode (AM) and high resolution (HR) (n = 33).

LAD, Left anterior descending coronary artery; LCx, Left Circumflex artery; RPD, Right posterior descending coronary artery; ** = p < 0.01; *** = p < 0.001, ns = not significant.

A movie of RTP-ASE images of a perfusion defect at stress is presented [see Additional file 1].

## Discussion

The results from the present study show overall similarities, but some differences, although no statistically significant, between the two different types of RTP power modulation techniques, thus data derived from different techniques for RTP-ASE are reasonably interchangeable. As judged by SPECT, there were highly significant agreements for both AM and HR in detecting ischemia. The finding that RTP can be used to accurately detect ischemia is in line with previous investigations [11-15,17,18]. Although there were no significant differences between AM and HR in agreement, there were some differences worth discussing.

Both methods showed high NPVs, which is of importance for correctly acquitting ischemia in patients. The NPV is especially important in this study cohort, since the prevalence of reversible ischemia was quite high (26%). Consequently, the value of PPV becomes relatively less important in this study. Wall motion abnormalities were present in 60% of the patients, indicating a very high prevalence of ischemic heart disease. However, the objective was to assess the accurate detection of reversible ischemia using AM and HR respectively, not to reveal the presence or absence of ischemic heart disease. Hence, any difference between AM and HR regarding PPVs becomes less important. Using SPECT as reference, HR showed higher values of kappa and accuracy, suggesting that HR is better suited than AM in this kind of population, i.e. with high pre-test probability of reversible myocardial ischemia. On the other hand, AM seems to have somewhat slightly higher sensitivity, which means that the risk of a false negative test values could actually be higher for HR. Sensitivity and specificity are somewhat connected to PPV and NPV, respectively, but are less affected by the prevalence of ischemia in the actual patient sample examined. Sensitivity and specificity values may, therefore, provide more reliable information about the precision of the test, rather than about the health of the patients. The finding that sensitivity figures were higher for AM than for HR indicates that AM RTP-ASE may be better at detecting the true reversibly ischemic patient, while the higher specificity values for HR might represent better identification of non-ischemic patients, as judged by SPECT. The slightly better sensitivity for AM might be due to the potentially easier visual assessment, to some point since echoes from contrast bubbles are displayed as colored pixels, in distinction to HR, where echoes are displayed in grey scale, but probably to a greater extent due to the lower resolution in AM. The lower resolution enables possible higher sensitivity because larger pixels provide more echo signal per pixel to process (higher statistical power) and thus a enhanced signal-to-noise ratio.

The higher kappa and accuracy values could therefore be due to superiority in correctly detecting non-ischemic territories, which were predominating in the present study population. The similarities and differences between AM and HR were approximately similar in the sole perfusion analysis, as compared with the combined perfusion and wall motion analysis. This indicates that agreement with SPECT can be maintained using wall motion analysis in coronary areas with perfusion artifacts, as previously suggested [15]. This should be viewed in relation to the results of non-interpretable coronary areas; where the high number of non-interpretable coronary areas in the sole perfusion analysis clearly illustrates lower feasibility, compared to the combined perfusion and wall motion analysis. Therefore, our results suggest that combined analysis is superior for maintaining good feasibility as well as good accuracy. It should be not that adenosine is not an optimal stressor concerning wall motion analysis. RTP-ASE is a highly available technique with possible bedside accessibility, which makes it appealing for clinical use, even in smaller hospitals with low access to angiographic or scintigraphic techniques.

### Study limitations

One obvious limitation is the subjectivity of RTP-ASE, since it is based on visual estimation of both perfusion and wall motion. Software tools for quantification of perfusion have been developed, which have shown promising results mainly in animal models [10,23,24] and a few clinical studies [16,25-29]. These results indicate that quantification of perfusion may soon be available for clinical use, but there is still need for larger clinical studies to evaluate feasibility. There are, however, need to improve user-friendly dedicated software, need for off-line analysis and the there is the additional cost of contrast agents.

It should be noted that this is an agreement study not an accuracy study. One limitation of the present investigation is that we cannot separate perfusion defects caused by macro-vascular or by micro-vascular disease because coronary angiography was not available in these patients. Further studies are needed to investigate the accuracy of RTP-ASE, SPECT, and perhaps Cardiac-MRI versus because coronary angiography.

Another limitation is that the RTP technique demands skilful operators and interpreters with a substantial amount of experience and knowledge in ultrasound physics, contrast agent characteristics and micro bubble behavior when exposed to ultrasound. However, this ultrasonic technique is still more available than other modalities and is useful for bedside evaluation, which still makes RTP-ASE an appealing and more accessible alternative.

The current study population consisted of patients with a high-risk for cardiovascular disease and this data cannot indiscriminately be used in low-risk patient-populations. Furthermore, the study results may obviously not be applicable to other types of populations with coronary artery disease, such as patients with acute coronary syndrome.

### Contrast safety

The U.S. Food and Drug Administration issued on October the 10th 2007 a "black box" warning for perflutren-containing contrast agents, which caused considerable controversies within the echocardiography community [30]. This warning was later relaxed [31]. Three recent large retrospective studies have disputed the suggestion that using the current generation echo contrast would pose a hazard to the patient [31-33]. Kusnetzky and co-workers reported single-centre data on 18.671 consecutive studies and found no increased acute mortality in patients who had received a contrast agent [32]. Main et al. reported data from a multicenter registry that included 4.300.966 consecutive patients [31]. Their finding was that patients who had received echo contrast actually had a lower mortality rate compared to those who had not received contrast. Furthermore, Dolan et al. compared 23.659 patients from three U.S. medical centres who had received echo contrast, at a rest examination, with 5.900 controls who had not received contrast, and found no increased mortality or nonfatal myocardial infarct in patients who had received contrast [33]. Dolan and co-workers extended their analysis and compared 10.788 patients who had undergone stress echocardiography (DSE or exercise stress echocardiography) and received contrast with 15.989 who had not received contrast. No increased mortality or nonfatal myocardial infarct in patients who had received contrast could be found also in this cohort. These studies clearly show that using echo contrast in stable patients does not pose a significant risk. This knowledge must be weighted against the hazards of a non-diagnostic echocardiography examination, and the potential risks accompanied by alternative tests. Finally, cost effectiveness remains to be demonstrated and real additive value of routine use of echo contrast in every patient that undergo stress echocardiography.

## Conclusion

The present investigation compared two different types RTP power modulation ASE. Both techniques showed highly significant agreements for the detection of reversible myocardial ischemia, using SPECT as method of reference. There was no significant difference between AM and HR in correctly detecting myocardial ischemia as judged by SPECT. This suggests that data derived from different techniques for RTP-ASE are reasonably interchangeable. When using the combined perfusion and wall motion interpretation, feasibility was higher, with almost no non-interpretable segments. Therefore, our results also suggest that combined analysis of perfusion and wall motion provides superior feasibility and accuracy of RTP-ASE.

## Competing interests

The authors declare that they have no competing interests.

## Authors' contributions

PG, RW, MD and RBW initiated the study. RW, MD and OT supervised the study and participated in the interpretation of the results and manuscript preparation. PG performed measurements, made all data conversions, plots and calculations from ultrasound data, and participated in the preparation of the manuscript. PG, KS, RW and MD participated in data collection, performed statistical analysis and participated in the interpretation of the results. LL and RBW participated in the interpretation of the results, in the creation of plots and in the preparation of the manuscript. MK participated in the interpretation of the results and preparation of the manuscript. All authors read and approved the final manuscript.

## Supplementary Material

Additional file 1 **Movies of RTP-ASE images at rest on the left hand side, and stress images on the right hand side. **Upper row represents angiomode modality and lower row represents grey scale images. Pleas note the apical lateral perfusion defect at stress. Click here for file
